# Optimizing Rearing of *Helicoverpa zea*: Impacts of Pupal Maturity, Emergence Synchrony, and Adult Cohort Size

**DOI:** 10.3390/insects17030342

**Published:** 2026-03-20

**Authors:** Shucong Lin, Tiago Silva, Bhavana Patla, Graham P. Head, Fangneng Huang

**Affiliations:** 1 Louisiana State University Agricultural Center, Baton Rouge, LA 70803, USA; slin@agcenter.lsu.edu (S.L.); tsilva@agcenter.lsu.edu (T.S.); bpatla@agcenter.lsu.edu (B.P.); 2 Bayer Crop Science, St. Louis, MO 63141, USA; graham.head@bayer.com

**Keywords:** bollworm, laboratory maintenance, mass rearing, mating success, reproduction

## Abstract

The bollworm/corn earworm, *Helicoverpa zea* (Boddie) (Lepidoptera: Noctuidae), is one of the most economically damaging crop pests in North America. Colonies of *H. zea* are notoriously difficult to maintain and frequently collapse in laboratory rearing. The persistent difficulty in maintaining healthy *H. zea* colonies has become a major obstacle to performing many research activities on the insect. To optimize colony maintenance, six populations were evaluated across three trials and six tests examining pupal maturity at diet removal, adult emergence synchrony, and cohort size at mating and reproduction. Females emerging from mature pupae produced more eggs than those from mid-aged (5–7 d) or young pupae (0–2 d). Synchronizing male and female emergence within one day yielded higher mating frequency, spermatophore transfer, and progeny, whereas a two-day difference reduced these metrics by 45–67%. Adult cohort size also influenced the outcomes, with ≥10 males and ≥10 females per cage enhancing reproductive success. Most matings occurred on nights 2–3, peaking within 2.5 h after lights off. Positive correlations were observed among mating frequency, spermatophore transfer, and progeny production. Overall, optimal performance was achieved by removing pupae only at maturity, synchronizing adult emergence within one day, and maintaining larger adult cohorts. These findings should establish key conditions to improve the mating success, reproduction, and laboratory rearing of *H. zea*.

## Simple Summary

The bollworm/corn earworm (*Helicoverpa zea*) is a major crop pest, but laboratory rearing challenges limit research progress. This study tested three selected biotic factors, pupal maturity, adult emergence synchrony, and adult cohort size that influence mating and reproduction. Results show that reproductive success is optimized when pupae are removed only at maturity, males and females emerge within one day of each other, and adult groups are kept large (≥10 of each sex per cage). These insights provide useful information to improve the mating, reproduction, and long-term laboratory colony maintenance of *H. zea*.

## 1. Introduction

The bollworm/corn earworm, *Helicoverpa zea* (Boddie) (Lepidoptera: Noctuidae), is one of the most economically damaging crop pests in North America [1,2,3]. This highly polyphagous species feeds on more than 200 host plants, including major crops such as maize, cotton, grain sorghum, and soybean [1]. Over the past three decades, transgenic cotton and maize expressing *Bacillus thuringiensis* (Bt) insecticidal proteins have been widely adopted in the United States to manage several lepidopteran pests, including *H. zea* [4,5,6].

The Bt proteins used in today’s commercial transgenic crops to manage lepidopteran pests can be categorized into three functional groups according to their modes of action: (1) Cry1 proteins (Cry1Ab, Cry1Ac, Cry1A.105, Cry1F); (2) Cry2 proteins (Cry2Ab2, Cry2Ae); and (3) Vip3Aa [7]. In the southern United States, cotton and maize are frequently grown in close proximity, and *H. zea* acts as a cross-crop pest of both crops [8]. As maize matures, *H. zea* typically disperses to other hosts, most notably cotton, grain sorghum, and soybean, allowing the pest to complete two to three additional generations each season [8,9]. Consequently, field populations in this region are likely exposed to multiple Bt proteins across successive generations [10,11], creating substantial challenges for the long-term sustainability of Bt crop technology. Indeed, widespread resistance to Bt cotton and maize expressing Cry proteins has been documented in *H. zea* populations in the United States, leaving Vip3Aa as the only Bt protein that remains fully effective against this pest [12,13,14,15,16]. These trends underscore the urgent need for effective insecticide resistance management (IRM) strategies to preserve the durability of Bt crops [6].

Reliable laboratory rearing is essential for research on insect biology, ecology, genetics, resistance evolution, and pest management. Several meridic diets, including WARD’s Stonefly Heliothis diet (Rochester, NY, USA) and the Southland diet (Lake Village, AR, USA), support larval development and are commonly used for *H. zea* rearing [15,17]. Nevertheless, *H. zea* colonies are notoriously difficult to maintain compared with many other lepidopteran species and frequently collapse under laboratory conditions due to poor mating success, low viable egg production, inbreeding, disease, or other unidentified factors [18,19,20,21]. In our recent work, numerous group-mating and single-pair mating attempts using adults derived from field-collected larvae resulted in low success in establishing subsequent generations, with single-pair matings being particularly unreliable [22,23]. Many field-collected populations failed to produce viable eggs during group mating in the laboratory.

The persistent difficulty in maintaining healthy *H. zea* colonies has become a major obstacle to advancing IRM research. Rearing challenges hinder essential activities such as F_2_ screens for resistance alleles [22,23], laboratory bioassays for resistance monitoring and baseline susceptibility estimation [18,19], and long-term colony maintenance [20]. Our review of the literature indicates that key biological parameters including calling, mating, and reproductive behaviors remain insufficiently characterized under laboratory conditions, likely contributing to ongoing rearing difficulties. Moreover, robust quantitative data linking specific biological traits to mating success and reproductive output in *H. zea* are still notably limited.

To address this knowledge gap, we conducted comprehensive studies examining how several key biotic and abiotic factors influence calling, mating, and reproduction in *H. zea*. The factors evaluated were selected based on years of laboratory rearing experience and previous findings, and included pupal maturity at removal from diet, male/female emergence synchrony, adult cohort size, larval cannibalism, presence of host plant tissue, and colony inbreeding [24]. Our objective was to identify conditions that enhance mating success and progeny production, thereby improving laboratory rearing and colony stability. In this paper, we report the effects of pupal maturity at removal from diet, male/female emergence synchrony, and adult cohort size on mating and reproduction under controlled laboratory conditions.

## 2. Materials and Methods

### 2.1. Sources of Helicoverpa zea Populations

To evaluate whether pupal maturity, emergence synchrony, and adult cohort size had consistent effects on mating and reproduction across different *H. zea* population sources, a total of six populations (denoted as BZ_Lab_, WB_18_, WB_19_, AL_21_, BR_23_, and BR_24_) were tested in three independent trials (Trial-I, Trial-II, and Trial-III) in this study (Table 1). BZ_Lab_ is a long-term laboratory population obtained from Benzon Research Inc. (Carlisle, PA, USA) and is known to be susceptible to Bt toxins and chemical insecticides [12,13,17]. Populations WB_18_ and WB_19_ originated from third- to fifth-instar larvae collected from non-Bt maize fields near Winnsboro, LA, USA during the 2018 and 2019 cropping seasons, respectively. AL_21_ is a Cry1A/Cry2A dual-protein Bt-resistant population established from fifth/sixth instars and pupae collected from ears of Genuity VT Double Pro^®^ maize near Alexandria, LA, USA in 2021. Genuity VT Double Pro^®^ is a widely planted Bt maize trait in the United States that expresses Cry1A.105 and Cry2Ab2 proteins for controlling aboveground lepidopteran pests, including *H. zea* [25]. Following field collection, AL_21_ underwent additional laboratory selections with Cry1A/Cry2A proteins, and diet-overlay bioassays confirmed high levels of resistance to both Cry1A.105 and Cry2Ab2 [26]. Population BR_23_ was initiated from third- to fifth-instar larvae collected from Genuity VT Double Pro^®^ maize fields near Baton Rouge, LA, USA in 2023, whereas BR_24_ was derived from larvae collected from non-Bt maize in 2024 at the same location.

As described above, extensive studies have documented widespread Cry1A/Cry2A resistance in *H. zea* populations throughout the southern United States, including all regions where insects for this study were sampled [12,13,14,15,16,27,28]. Therefore, even populations collected from non-Bt maize fields (such as WB_18_, WB_19_, and BR_24_) were likely resistant to Cry proteins. In contrast, BZ_Lab_ served as a susceptible reference population and has been widely used in resistance-monitoring bioassays [12,13,17]. To establish the populations for this study, field-collected larvae and BZ_Lab_ were reared in 30-mL cups (SOLO, Chicago, IL, USA) containing WARD’s Stonefly Heliothis diet (Rochester, NY, USA). Rearing temperatures were adjusted as needed to synchronize development within each population, following the procedures described in reference [17].

### 2.2. Effect of Pupal Maturity on Reproduction of Helicoverpa zea (Trial-I)

Three *H. zea* populations (BZ_Lab_, WB_18_, and WB_19_) served as the insect sources for Trial-I. Under normal room conditions (~24–26 °C), *H. zea* adults typically emerge 12–14 days after pupation [29]. Trial-I assessed three pupal maturity classes for each population, defined by the interval between pupation and removal from diet for placement into cages for adult emergence, mating, and reproduction. These age classes were newly pupated (<2 days old), middle-aged (5–7 days old), and mature (pre-emergence). Trial-I was conducted in summer 2019. At that time, WB_18_ had been maintained in laboratory culture for approximately one year (F8) following the rearing procedures described in reference [17]. To generate pupae of known age, neonates of BZ_Lab_ and WB_18_ were individually reared in 30-mL cups containing ~8 g of diet. For WB_19_, field-collected larvae were reared individually using the same diet, and the resulting pupae (F0) were used directly in the experiment.

To obtain pupae of the designated age classes, pupation was monitored daily beginning with the appearance of the first pupa in each population. When most larvae had pupated, individuals were sexed by examining the position of the genital opening on the underside of the abdomen [30] and separated by both sex and pupation time. Following sexing, female and male pupae were either transferred immediately into 20-L cylindrical cages (27–31 cm diameter × 35 cm height, Safco Products Co., New Hope, MN, USA) at a density of 15 females and 15 males per cage for the newly pupated group or returned to the diet to continue development. Five days later, the middle-aged group (15 females and 15 males per cage) was transferred to cages. The mature group (15 females and 15 males per cage) was collected and caged when the first adult moth emerged in any rearing cup of that population.

The 20-L cage design used for adult emergence, mating, and oviposition followed the method described in reference [17]. Cages containing pupae of different age classes were arranged in a randomized complete block design (RCBD) within an environmentally controlled rearing room maintained at 26 °C, >70% relative humidity, and a 14:10 h (L:D) photoperiod. Each combination of population and pupal maturity included four replications (blocks), with one cage per replication (15 females and 15 males). To satisfy the RCBD structure, the rearing room was divided into four sections, each serving as a block. Eggs deposited on the gauze cloth covering each cage were collected daily and counted by visual inspection following the procedures of reference [31].

### 2.3. Effect of Male/Female Emergence Synchrony on Mating and Reproduction of Helicoverpa zea (Trial-II)

Trial-II consisted of three independent tests, each conducted with a different *H. zea* population: AL_21_, BR_23_, or BR_24_. Results from Trial-I demonstrated that adults emerging from mature pupae that were kept on the rearing diet until eclosion produced more eggs than adults originating from younger pupae (see Results). In addition, preliminary observations indicated that the presence of larval cannibalism during diet-based rearing and inclusion of host plant tissue in adult cages enhanced mating success and progeny production [32]. Consequently, all pupae used in Trial-II were maintained in diet until maturity, and all three tests were conducted under conditions that promoted larval cannibalism and were provided with host plant tissue (maize ears with husks and silks) inside the adult cages.

*Helicoverpa zea* larvae exhibit cannibalistic behavior beginning in the second instar, with aggression and frequency increasing substantially by the third instar [33]. To induce cannibalism in Trial-II, three neonates from each population (post–field collection and, for AL_21_, following Bt-resistance selection) were placed together in 30-mL cups containing approximately 8 g of diet. Cups were arranged in 30-well trays and maintained in environmental chambers at 26 °C, ~50% relative humidity, and a 16:8 h light:dark photoperiod until pupation. Mature pupae were removed from cups containing only a single surviving pupa and transferred to 20-L cages following the procedures described for Trial-I. Any larval rearing cups containing more than one pupa were discarded. The AL_21_ test was conducted from April to May 2024; prior to testing, AL_21_ had been maintained in the laboratory for 20 generations (~3 years). The BR_23_ test was conducted from May to June 2024, and this population had been reared under the same laboratory conditions for six generations before use. The BR_24_ test was conducted in August 2024 using F1 progeny of field-collected larvae.

Each test evaluated mating and reproduction under five female/male adult-emergence synchronies: (1) newly emerged (<14 h old) virgin females paired with newly emerged virgin males (F_0d_M_0d_); (2) 1-day-old virgin females paired with newly emerged virgin males (F_1d_M_0d_); (3) newly emerged virgin females paired with 1-day-old virgin males (F_0d_M_1d_); (4) 2-day-old virgin females paired with newly emerged virgin males (F_2d_M_0d_); and (5) newly emerged virgin females paired with 2-day-old virgin males (F_0d_M_2d_). Female and male pupae were placed into separate 20-L cages until adult emergence.

Upon emergence, 10 females and 10 males of the designated ages were transferred into a 20-L cage provisioned with vermiculite and a honey-water solution, as described previously [17]. A fresh R1-stage maize ear with husk and silks, positioned upright in a 266-mL plastic cup containing water-saturated paper towel, was also placed inside each cage. Cages were arranged in an RCBD with four blocks in the same environmentally controlled rearing room used for Trial-I. After cage placement, the room lights were turned off, and a 10:14 h (D:L) photoperiod was maintained for the duration of the tests.

Mating pairs were recorded by manual visual observation using a red flashlight beginning 30 min after lights off and at 2-h intervals throughout each nocturnal period for six consecutive nights. Eggs were collected daily from each cage and counted by visual inspection. Egg-hatching rate for each replication was determined by counting the total number of eggs and the number of unhatched eggs. Progeny production was calculated as the product of total eggs laid and the corresponding hatching rate. After all adults in a cage had died, all females were preserved in 70% ethanol and later dissected under a microscope to count spermatophores present in the bursa copulatrix [34].

### 2.4. Effect of Cohort Size in Adult Cages on Mating and Reproduction of Helicoverpa zea (Trial-III)

Two independent tests were conducted in Trial-III, each using a different *H. zea* population: AL_21_ and BR_24_. The AL_21_ test was performed in July 2024; prior to testing, this population had been maintained under laboratory conditions for 22 generations (approximately 3 years). In contrast, the BR_24_ test evaluated the F2 generation derived directly from the field collections and was conducted in October 2024. Results from Trial-II (population AL_21_) indicated that among the five adult-emergence synchronies examined, the F_1d_M_0d_ and F_0d_M_1d_ treatments produced the highest progeny output (see Results). Furthermore, previous studies have shown that maintaining at least 10 males and 10 females per adult cage is sufficient to support normal mating behavior and colony productivity [13,14,15,30]. Building on these findings, Trial-III aimed to identify the minimum adult cohort size that did not compromise mating success or reproductive performance. Accordingly, three adult cohort sizes (5♀ × 5♂, 10♀ × 10♂, and 20♀ × 20♂) were evaluated under the two emergence-synchrony treatments, F_1d_M_0d_ and F_0d_M_1d_.

As described for Trial-II, larvae from each population were reared in groups of three per 30-mL cup to promote cannibalism and maintained on diet until maturity. Mature pupae were removed from the diet, sexed, and placed into separate 20-L cages until adult emergence. After emergence, the designated numbers of appropriately aged females and males were introduced into each 20-L cage, along with a maize ear prepared as described in Trial-II. Cages were arranged in an RCBD with four replications for each combination of emergence synchrony and adult cohort size. Mating observations during nocturnal periods, daily egg collection, egg-hatching rate, progeny production, and spermatophore counts in dissected females were recorded following the same procedures used in Trial-II.

### 2.5. Data Analysis

To satisfy the assumption of normality required for statistical analyses, the original data for egg production, progeny production, number of mating occurrences over six nights, and number of spermatophores per female were log-transformed using log (x + 1) for each treatment replication. Transformed egg-production data from Trial-I were analyzed using two-way analysis of variance (ANOVA) (SAS 9.4 PROC GLM), with insect population and pupal maturity as the two main factors [35].

For Trial-II, transformed data on mating occurrences, spermatophore transfer, and progeny production were first analyzed separately for each of the three tests, with adult-emergence synchrony as the treatment factor. To draw broader conclusions across populations, data from the three independent tests were then pooled and analyzed using one-way ANOVA with insect population (test) and replication treated as random effects (SAS 9.4 PROC MIXED) [35].

Similarly, transformed data from Trial-III, including mating occurrences, spermatophore counts, and progeny production, were first analyzed separately for each of the two tests using two-way ANOVA, with adult-emergence synchrony and adult cohort size as the main factors. The datasets from the two tests were then pooled and analyzed with test (insect population) and replication as random factors (SAS 9.4 PROC MIXED) [35]. For all ANOVA, treatment means were separated using Tukey’s HSD test at α = 0.05 and untransformed means are presented in figures.

In both Trial-II and Trial-III, in addition to quantifying the mean number of spermatophores transferred to females, we calculated the proportion of females containing at least one spermatophore and the proportion containing two or more. For each trial, regression analyses (SAS 9.4 PROC REG) were performed to assess the linear relationships among three key biological parameters, number of mating occurrences, mean number of spermatophores received per female, and progeny production, as well as between the percentages of females receiving at least one or at least two spermatophores and the corresponding progeny production. To characterize temporal patterns of mating activity, we calculated the percentage of total mating occurrences attributable to each observation time for each treatment. This was determined by dividing the number of mating events recorded across the four replicates at a given observation by the total number of mating events documented over the six observation nights for that treatment. Histograms were generated to visualize the distribution of percentage mating occurrence for each treatment in both trials. Additionally, the mating–frequency datasets from the three tests in Trial-II were combined and analyzed using a two-way ANOVA with adult-emergence synchrony and night as the two main factors, and insect population (test) and replication treated as random factors (SAS 9.4 PROC MIXED) [35].

## 3. Results

### 3.1. Effect of Pupal Maturity on Reproduction of Helicoverpa zea

**Figure 1:**
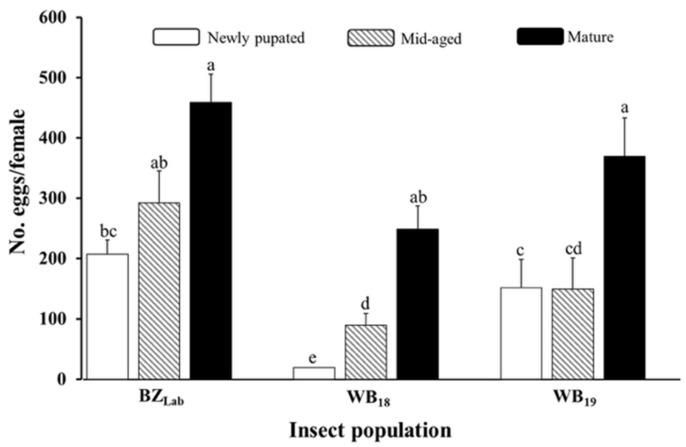
Progeny production (mean ± SEM) of three *Helicoverpa zea* populations (BZ_Lab_, WB_18_, and WB_19_) reared with different pupal maturities at removal from diet (Trial-I). Mean values followed by the same letter were not significantly different (ANOVA, Tukey’s HSD test, α = 0.05).

The effects of insect population, pupal maturity, and their interaction on egg production in Trial-I were all significant (*F*_2,19_ = 34.78, *p* < 0.0001 for population; *F*_2,19_ = 39.22, *p* < 0.0001 for pupal maturity; and *F*_4,19_ = 5.89, *p* = 0.0029 for interaction). Egg production of moths emerging from mature (pre-emergence) pupae at removal from diet did not differ significantly among the three populations (*p* > 0.05), ranging from 248.9 eggs for WB_18_ to 459.4 eggs for BZ_Lab_ (Figure 1). In contrast, egg production was significantly reduced (*p* < 0.05) when pupae were removed from the diet at younger stages. The magnitude of reduction was greater for the two populations originating from field-collected larvae (WB_18_ and WB_19_) than for the laboratory-reared population (BZ_Lab_). For example, relative to moths emerging from mature pupae, those emerging from newly pupated individuals produced 92.1% and 58.9% fewer eggs for WB_18_ and WB_19_, respectively, whereas the reduction for BZ_Lab_ was 54.9%. A similar pattern was observed for pupae removed at the middle-aged stage across all three populations (Figure 1).

### 3.2. Effect of Male/Female Emergence Synchrony on Mating and Reproduction of Helicoverpa zea

**Figure 2:**
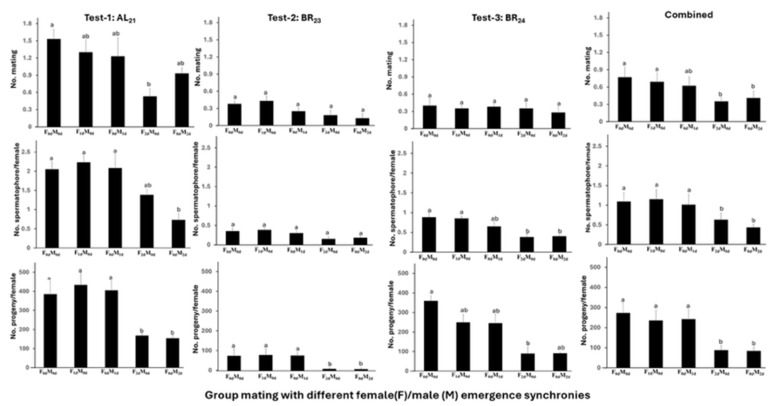
Mating frequency, spermatophore number, and progeny production (mean ± SEM) of three *Helicoverpa zea* populations (AL_21_, BR_23_, and BR_24_) under different male/female emergence synchronies (Trial II). Mean values followed by the same letter were not significantly different (ANOVA, Tukey’s HSD test, α = 0.05).

Although the BR_23_ test in Trial-II yielded lower absolute values for mating frequency, spermatophore transfer, and progeny production than the AL_21_ and BR_24_ tests, the overall patterns were consistent across all three populations (Figure 2). The effect of male/female emergence synchrony on number of matings was not significant for Test-2 (*F*_4,12_ = 3.18, *p* = 0.0534) and Test-3 (*F*_4,12_ = 1.28, *p* = 0.3315), but significant for Test-1 (*F*_4,12_ = 4.50, *p* = 0.0188) and the combined data (*F*_4,44_ = 5.70, *p* = 0.0009). Based on the combined dataset, treatments in which males and females emerged on the same day or within one day (F_0d_M_0d_, F_1d_M_0d_, F_0d_M_1d_) produced 0.62–0.77 matings per female, whereas treatments with a two-day emergence difference (F_2d_M_0d_, F_0d_M_2d_) produced only 0.35–0.41 matings per female (Figure 2).

**Figure 3:**
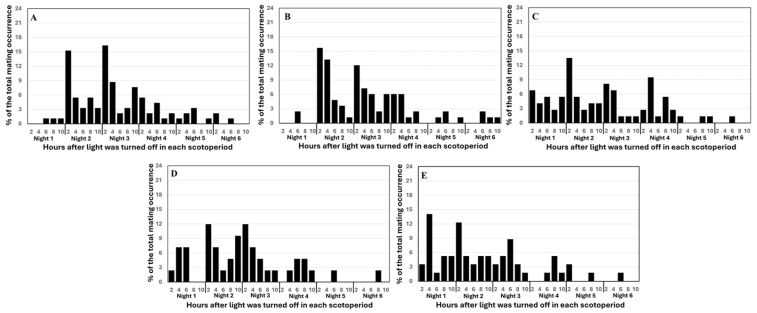
Mating occurrence across observation periods for three *Helicoverpa zea* populations (AL_21_, BR_23_, and BR_24_) reared with different pupal maturities at removal from diet (Trial II). Percent occurrence at each observation was calculated as the number of mating pairs recorded across four replications divided by the total number of mating pairs observed over six nights. (**A**): F_0d_M_0d_—ten newly emerged virginal females and ten newly emerged virginal males; (**B**): F_1d_M_0d_—ten 1-d old virginal females and ten newly emerged virginal males, (**C**): F_0d_M_1d_—ten newly emerged virginal females and ten 1-d old virginal males, (**D**): F_2d_M_0d_—ten 2-d old virginal females and ten newly emerged virginal males, and (**E**): F_0d_M_2d_—ten newly emerged virginal females and ten 2-d old virginal males.

Temporal patterns of mating also differed among emergence-synchrony treatments (Figure 3). Two-way ANOVA showed that the main effect of emergence synchrony on mating frequency was not significant (*F*_4,226_ = 0.13, *p* = 0.9731), but the effects of night and the interaction were significant (*F*_5,226_ = 28.54, *p* < 0.0001 for synchrony and *F*_20,226_ = 2.54, *p* < 0.0005 for interaction). In the treatments where females emerged at the same time or earlier than males (F_0d_M_0d_, F_1d_M_0d_, F_2d_M_0d_), most mating occurred during nights 2 and 3. In contrast, when males emerged earlier (F_0d_M_1d_, F_0d_M_2d_), most mating occurred during nights 1–3 (Figure S1). Across all treatments, nightly mating peaked within the first 2.5 h after lights-off, and few mating events occurred after night 4 (Figure 3).

Patterns of spermatophore transfer mirrored those for mating frequency. The effect of emergence synchrony on spermatophore transfer was not significant for Test-2 (*F*_4,12_ = 2.50, *p* = 0.0984), but significant for Test-1 (*F*_4,12_ = 12.45, *p* = 0.0003), Test-3 (*F*_4,12_ = 6.68, *p* = 0.0046), and combined data (*F*_4,44_ = 13.87, *p* < 0.0001). Based on the combined data, 57.5–66.7% of females in the three synchronized treatments (F_0d_M_0d_, F_1d_M_0d_, F_0d_M_1d_) received at least one spermatophore, with an average of 1.01–1.15 per female (Figure 2; Supplementary Table S1). Approximately 30.5% of females received multiple spermatophores (up to five). In contrast, only 35.0–43.3% of females in the two asynchronous treatments (F_2d_M_0d_, F_0d_M_2d_) received at least one spermatophore (up to four), with averages of 0.43–0.63 per female; only 11.3% received multiple spermatophores (Supplementary Table S1).

Progeny production was significantly affected by emergence synchrony in all three tests (*F*_4,12_ = 21.79, *p* < 0.0001 for Test-1; *F*_4,12_ = 21.37, *p* < 0.0001 for Test-2; and *F*_4,12_ = 3.64, *p* = 0.0366 for Test-3) and in the combined analysis (*F*_4,44_ = 16.34, *p* < 0.0001). Females in the synchronized treatments (F_0d_M_0d_, F_1d_M_0d_, F_0d_M_1d_) produced 241.7–272.2 progeny per female, whereas those in the asynchronous treatments (F_2d_M_0d_, F_0d_M_2d_) produced only 83.3–88.0 progeny per female (Figure 2).

Regression analyses across all treatments and tests revealed strong positive linear relationships among mating frequency, spermatophore transfer, and progeny production (Table 2).

### 3.3. Effect of Cohort Size in Adult Cages on Mating and Reproduction of Helicoverpa zea

**Figure 4:**
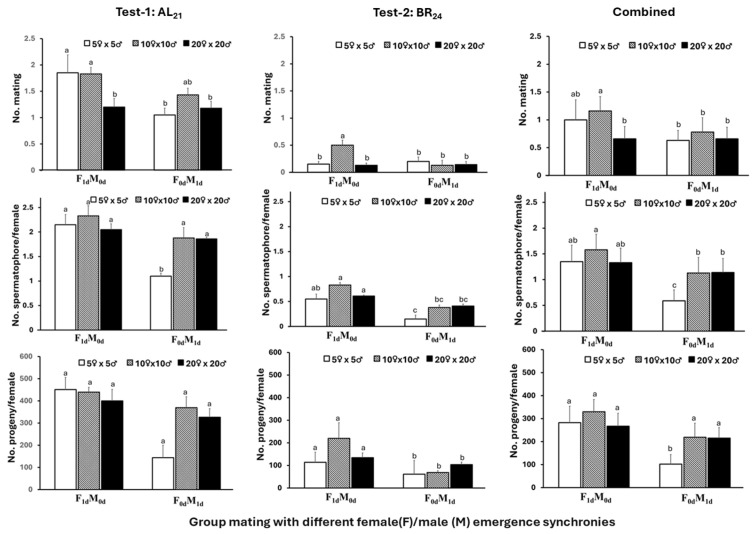
Mating frequency, spermatophore number, and progeny production (mean ± SEM) of two *Helicoverpa zea* populations (AL_21_, BR_24_) under two different male/female emergence synchronies and three cohort sizes in adult cages (Trial III). Mean values followed by the same letter were not significantly different (ANOVA, Tukey’s HSD test, α = 0.05).

**Figure 5:**
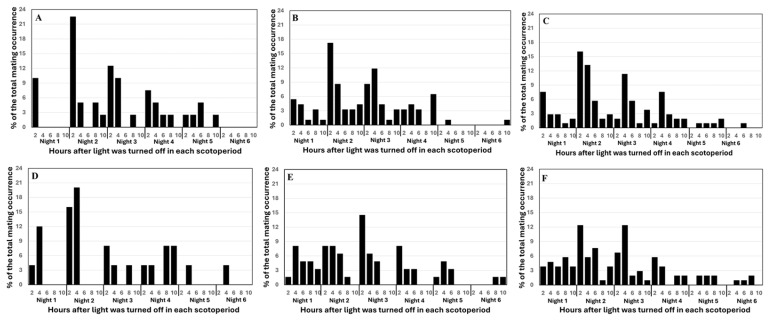
Mating occurrence across observation periods and two *Helicoverpa zea* populations under two different male/female emergence synchronies and three cohort size in adult cages (Trial III). Percent occurrence at each observation was calculated as the number of mating pairs recorded across four replications and the two insect populations divided by the total number of mating pairs observed over six nights. (**A**): Five 1-d old virginal females and five newly emerged virginal males, (**B**): Ten 1-d old virginal females and ten newly emerged virginal males, (**C**): Twenty 1-d old virginal females and twenty newly emerged virginal males, (**D**): Five newly emerged virginal females and five 1-d old males, (**E**): Ten newly emerged virginal females and Ten 1-d old males, and (**F**): Twenty newly emerged virginal females and twenty 1-d old males.

Substantial variation also occurred between the two tests of Trial-III. In Test-1, both emergence synchrony and adult cohort size had significant effects on the number of matings (*F*_1,15_ = 10.19, *p* = 0.0061 for synchrony and *F_2_*_,15_ = 4.46, *p* = 0.0302 for cohort size), whereas their interaction was not significant (*F*_2,15_ = 3.14, *p* = 0.0726). In Test-2, synchrony alone was not significant (*F*_1,15_ = 4.19, *p* = 0.0586), but cohort size (*F*_2,15_ = 4.04, *p* = 0.0396) and the interaction (*F*_2,15_ = 7.06, *p* = 0.0069) were significant. Analysis of the combined dataset showed significant effects of emergence synchrony (*F*_1,35_ = 11.73, *p* = 0.0016), cohort size (*F*_2,35_ = 6.83, *p* = 0.0031), and their interaction (*F*_2,35_ = 3.45, *p* = 0.0436). Based on the combined data, the highest mating frequency (1.16 matings per female) occurred in the F_1d_M_0d_ treatment with 10♀ × 10♂, which did not differ significantly from the F_1d_M_0d_ treatment with 5♀ × 5♂ but exceeded all other treatment combinations (Figure 4). Temporal mating patterns resembled those in Trial-II, with most mating occurring during the first four nights and peak activity within the first 2.5 h after lights-off on nights 2 and 3 (Figure 5).

The number of spermatophores transferred from males to females in Test-1 was significantly affected by emergence synchrony (*F*_1,15_ = 29.13, *p* < 0.0001), cohort size (*F*_2,15_ = 8.62, *p* = 0.0032), and their interaction (*F*_2,15_ = 7.38, *p* = 0.0059). In Test-2, both main effects were significant (*F*_1,15_ = 28.84, *p* < 0.0001 for synchrony and *F*_2,15_ = 5.85, *p* = 0.0132 for cohort size), whereas the interaction was not significant (*F*_2,15_ = 1.53, *p* = 0.2487). Analysis of the combined dataset showed significant effects of emergence synchrony (*F*_1_,_35_ = 90.46, *p* < 0.0001), cohort size (*F*_2,35_ = 23.61, *p* < 0.0001), and their interaction (*F*_2,35_ = 11.68, *p* = 0.0001). Based on combined data across the two tests, females in the F_1d_M_0d_ treatments received an average of 1.33–1.58 spermatophores across the three cohort sizes, and 38.2% received more than one (up to five). In contrast, spermatophore numbers received per female declined in the F_0d_M_1d_ treatments, particularly in the 5♀ × 5♂ cohort, which averaged only 0.59 spermatophores per female (up to two) (Figure 4; Supplementary Table S2).

Notable differences between the two tests were also evident in progeny production. In Test-1, progeny output was not significantly affected by emergence synchrony (*F*_1,15_ = 3.65, *p* = 0.0753), cohort size (*F*_2,15_ = 1.92, *p* = 0.1806), or their interaction (*F*_2,15_ = 2.16, *p* = 0.1495). In contrast, both main effects were significant in Test-2 (*F*_1,15_ = 8.89, *p* = 0.0093 for synchrony and *F*_2,15_ = 6.54, *p* = 0.0091 for cohort size), whereas the interaction was not (*F*_2,15_ = 3.16, *p* = 0.0718). Analysis of the combined dataset revealed significant effects of emergence synchrony (*F*_1,35_ = 12.94, *p* = 0.0010), cohort size (*F*_2,35_ = 8.41, *p* = 0.0010), and their interaction (*F*_2,35_ = 5.57, *p* = 0.0079). Progeny production was consistently higher in F_1d_M_0d_ than in F_0d_M_1d_; the difference was significant (*p* < 0.05) for the 5♀ × 5♂ cohort but not for the larger cohort sizes (Figure 4).

Regression analyses again revealed strong positive linear relationships among mating frequency, spermatophore transfer, and progeny production (Table 3).

## 4. Discussion

The superior reproductive performance observed in *H. zea* populations when pupae were removed from the diet at the mature stage, relative to those removed earlier, likely reflects the species’ natural pupation ecology. Under field conditions, *H. zea* larvae feed on aboveground plant tissues and, upon reaching maturity, leave the host plant and drop to the soil to pupate. Pupae remain within the soil matrix until adult emergence [36,37,38]. Pupae that remain embedded in the diet therefore experience a microhabitat more similar to these soil-like conditions than the comparatively artificial environment of the adult cage. This closer alignment with their natural developmental setting may underlie the enhanced reproductive performance observed in mature-pupae treatments. Accordingly, for laboratory rearing, pupae should be maintained within the diet until they reach the mature stage, and the removal of younger pupae for placement into adult cages should be avoided.

Consistent with patterns reported for other lepidopteran species [39,40,41,42], our results show that pairing 2-day-old males and females (e.g., F_2d_M_0d_ or F_0d_M_2d_) markedly reduces mating success and progeny production in *H. zea*. As shown in the current study, newly emerged virgin adults rarely copulated during the first scotophase, with most mating events occurring on the second and third nights. Consequently, pairing 1-day-old males and females (e.g., F_1d_M_1d_) is unlikely to impair reproductive output, whereas a two-day delay substantially diminishes fecundity. Age-related effects on reproduction are well-documented across Lepidoptera [43,44,45]. In males, advancing age is associated with reduced spermatophore quality and diminished transfer efficiency [46], while delayed mating in females of species such as *Lymantria dispar*, *Cnaphalocrocis medinalis*, and *Spodoptera litura* leads to decreased pheromone production and lower egg viability [47,48,49]. Under laboratory conditions, *H. zea* adults typically exhibit short lifespans (~6 days) [9,31], likely rendering them particularly sensitive to aging effects and explaining the pronounced reduction in fecundity following only a two-day delay in mating. In field populations and within laboratory colonies, females generally emerge approximately one day earlier than males [50,51]. The enhanced mating success and reproductive output observed when male and female emergence was synchronized, relative to asynchronous emergence, indicate that maintaining natural emergence patterns is critical for optimal colony performance. Collectively, these findings highlight the importance of preserving emergence synchrony to support effective rearing and the long-term maintenance of *H. zea* laboratory colonies.

The presence of a spermatophore within the reproductive tract of a lepidopteran female is widely recognized as evidence of successful copulation [52]. In this study, the consistent positive association between the number of observed matings and the number of spermatophores recovered from the bursa copulatrix across all trials indicates that spermatophore counts provide a reliable proxy for mating frequency in *H. zea* under laboratory conditions. This conclusion aligns with findings for several other lepidopteran species [39,46,53,54,55]. Despite this strong correspondence, the number of matings directly observed in this study was generally lower than the number of spermatophores detected. *Helicoverpa zea* copulation can last 1–2 h [56], thus, the two-hour observation interval used in this study may have been insufficient to capture all mating events, leading to underestimation of the actual mating frequency.

Consistent with patterns documented in other lepidopteran species [39,57,58,59,60], our findings reveal a strong positive association between the mean number of spermatophores transferred and progeny production in *H. zea*. Multiple mating events were frequent, and repeated successful copulations were consistently linked to increased reproductive output. The pronounced positive relationships among mating frequency, spermatophore transfer, and progeny production likely reflect cumulative benefits of repeated mating, through which females acquire greater quantities of sperm as well as accessory gland substances contained within spermatophores, compounds known to stimulate oviposition and enhance fertilization efficiency [61]. The strong concordance among mating frequency, spermatophore transfer, and progeny production indicates that any ecological or behavioral factors that promote increased mating activity are likely to enhance spermatophore transfer and, consequently, elevate progeny production.

In summary, despite some variability among insect populations and across tests, results from the three trials of this study demonstrated that pupal maturity at removal from diet, male/female emergence synchrony, and adult cohort size significantly influenced mating success and progeny production in *H. zea* under laboratory conditions. Females emerging from mature pupae produced substantially more eggs than those derived from younger pupae. Synchronization of male and female adult emergence within a one-day interval yielded higher mating frequency, increased spermatophore transfer, and greater progeny output compared with pairs differing by two days. Similarly, reducing adult cohort size to five pairs per cage could diminish mating activity, spermatophore transfer, and progeny production relative to cohorts of 10 or 20 pairs. Across the trials and tests, most matings of *H. zea* moths occurred on nights 2–3, with peak activity concentrated within the first 2.5 h after lights off. Strong positive correlations were observed among mating frequency, spermatophore transfer, and progeny production. Collectively, these findings indicate that optimal reproductive performance should be achieved by (1) removing pupae from diet only at the mature stage, (2) synchronizing adult male and female emergence within one day, and (3) maintaining relatively large adult cohorts (≥10 males and ≥10 females per cage). These results provide critical information for enhancing mating success and reproduction in *H. zea*, thereby improving the laboratory rearing protocols and colony maintenance of the insect.

## Figures and Tables

**Table 1:** Sources of six *Helicoverpa zea* populations used in the study.

Population ID	Sources	Used in
BZ_Lab_	A long-term laboratory-maintained population obtained from Benzon Research Inc. (Carlisle, PA, USA).	Trial-I
WB_18_	A population initiated from 283 larvae collected from non-Bt maize fields near Winnsboro, LA, USA in 2018. Before it was used in this study, WB_18_ had been reared in the laboratory for one year (8 generations).	Trial-I
WB_19_	A population originated from 950 larvae collected from non-Bt maize fields at the same location as WB_18_ in 2019. Pupae/adults (F0) developed from the field-collected larvae were directly used in this study.	Trial-I
AL_21_	A dual-protein Cry1A/Cry2A resistant population initiated from >150 larvae and pupae from Genuity VT Double Pro^®^ maize planted near Alexandria, LA, USA in 2021. The offspring of the field-collected population were further selected against Cry1A/Cry2A proteins as described in [26]. AL_21_ had been maintained in the laboratory for 20 and 22 generations before it was used in this study in Trial-II and Trial-III, respectively.	Trial-II, Trial-III
BR_23_	A population initiated from ~180 larvae collected from Genuity VT Double Pro^®^ maize near Baton Rouge, LA, USA in 2023. BR_23_ had been reared in laboratory conditions for six generations before it was used in this study.	Trial-II
BR_24_	A population originated from ~240 larvae collected from non-Bt maize fields near Baton Rouge, LA, USA in 2024. BR_24_ had been reared under laboratory conditions for one and two generations before it was used in this study in Trial-II and Trial-III, respectively.	Trial-II, Trial-III

**Table 2:** Linear relationships among number of mating occurrences, frequency of spermatophores, and progeny production in Trial-II.

Parameter	Regression Equation	*R* ^2^	*p*-Value
Mean number of mating (X) versus spermatophores received per female (Y)	Y = 0.05 + 1.45X	0.82	<0.0001
Mean number of mating (X) versus progeny production (Y)	Y = 42.6 + 257.0X	0.62	<0.0001
No. spermatophore transfer (X) versus progeny production (Y)	Y = 29.6 + 182.7X	0.80	<0.0001
% of ≥1 spermatophore received per female (X) versus progeny production (Y)	Y = −71.9 + 481.0X	0.86	<0.0001
% of ≥2 spermatophore received per female (X) versus progeny production (Y)	Y = 101.4 + 378.4X	0.53	0.0020

**Table 3:** Linear relationships among number of mating occurrences, frequency of spermatophores, and progeny production in Trial-III.

Parameter	Regression Equation	*R* ^2^	*p*-Value
Mean number of mating (X) versus spermatophores received per female (Y)	Y = 0.27 + 1.13X	0.92	<0.0001
Mean number of mating (X) versus progeny production (Y)	Y = 68.5 + 205.2X	0.86	<0.0001
No. spermatophore transfer (X) versus progeny production (Y)	Y = 17.6 + 183.2X	0.95	<0.0001
% of ≥1 spermatophore received per female (X) versus progeny production (Y)	Y = −93.6 + 463.9X	0.80	<0.0001
% of ≥2 spermatophore received per female (X) versus progeny production (Y)	Y = 102.3 + 409.8X	0.90	<0.0001

## Data Availability

The original contributions presented in this study are included in the article/Supplementary Material. Further inquiries can be directed to the corresponding author.

## Supplementary Materials

The following supporting information can be downloaded at: https://www.mdpi.com/article/10.3390/insects17030342/s1, Table S1: Frequency (%) of spermatophores observed in each female of the three *H. zea* populations, AL_21_, BR_23_, and BR_24_ with different aged females and males in group mating conditions (Trial-II); Table S2: Frequency (%) of spermatophores observed in each female of the two *H. zea* populations, AL_21_ and BR_24_ with different aged adult moths and population size in group mating conditions (Trial-III); Figure S1: Mating occurrence across six nights under group-mating conditions with different female (F) and male (M) emergence synchronies (Trial II). Percent occurrence (mean ± SEM) for each night was calculated as the number of mating pairs observed during the five nightly observation periods divided by the total number of mating events recorded over all six nights. Replications with two or fewer mating events during the six-night observation period were excluded from the data analysis. Mean values sharing a same letter are not significantly different (ANOVA, Tukey’s HSD, α = 0.05). Only the first and the last letters are presented over the bar if four or more letters were needed for the HSD tests. For example, the label of a-e over the bar representing the mean of ‘Night 5’ of F_0d_M_0d_ means abcde.
