# Supplementary material for: Optimizing Rearing of Helicoverpa zea: Impacts of Pupal Maturity, Emergence Synchrony, and Adult Cohort Size
Source: Insects. 2026 Mar 20;17(3):342. doi: 10.3390/insects17030342 (PMC13026774; doi:10.3390/insects17030342)
Supplement: Supplementary file 1 [file insects-17-00342-s001.zip › Supporting Information.pdf]

Supporting Information: Table S1, Table S2, and Figure S1

Optimizing Rearing of *Helicoverpa zea*: Impacts of Pupal Maturity, Emergence Synchrony, and Adult Cohort Size

Shucong Lin [1], Tiago Silva [1], Bhavana Patla [1], Graham P. Head [2] and Fangneng Huang [1,*]

[1] Louisiana State University Agricultural Center, Baton Rouge, LA 70803, USA; slin@agcenter.lsu.edu (S.L.); tsilva@agcenter.lsu.edu (T.S.); bpatla@agcenter.lsu.edu (B.P.)

[2] Bayer Crop Science, St. Louis, MO 63141, USA; graham.head@bayer.com

* Correspondence: fhuang@agcenter.lsu.edu

Supporting Information Table S1. Frequency (%) of spermatophores observed in each female of the three *Helicoverpa zea* populations, AL_{21}, BR_{23}, and BR_{24} with different aged females and males in group mating conditions (Trial-II).

No. spermatophores /female	$F_{0d}M_{0d}$	$F_{1d}M_{0d}$	$F_{0d}M_{1d}$	$F_{2d}M_{0d}$	$F_{0d}M_{2d}$
		AL_{21}			
0	7.5	2.5	7.5	17.5	50.0
1	20.0	22.5	17.5	40.0	32.5
2	47.5	35.0	47.5	32.5	12.5
3	12.5	30.0	20.0	7.5	5.0
4	10.0	10.0	2.5	2.5	0.0
5	2.5	0.0	5.0	0.0	0.0
		BR_{23}			
0	70.0	70.0	72.5	87.5	82.5
1	25.0	25.0	25.0	10.0	17.5
2	5.0	5.0	2.5	2.5	0.0
3	0.0	0.0	0.0	0.0	0.0
4	0.0	0.0	0.0	0.0	0.0
5	0.0	0.0	0.0	0.0	0.0
		BR_{24}			
0	30.0	27.5	47.5	65.0	62.5
1	55.0	60.0	40.0	32.5	35.0
2	12.5	12.5	12.5	2.5	2.5
3	2.5	0.0	0.0	0.0	0.0
4	0.0	0.0	0.0	0.0	0.0
5	0.0	0.0	0.0	0.0	0.0
		Pooled			
0	35.8	33.3	42.5	56.7	65.0
1	33.3	35.8	27.5	27.5	28.3
2	21.7	17.5	20.8	12.5	5.0
3	5.0	10.0	6.7	2.5	1.7
4	3.3	3.3	0.8	0.8	0.0
5	0.8	0.0	1.7	0.0	0.0

Supporting Information Table S2. Frequency (%) of spermatophores observed in each female of the two *Helicoverpa zea* populations, AL21 and BR24 with different aged adult moths and population size in group mating conditions (Trial-III).

	$F_{1d}M_{0d}$			$F_{0d}M_{1d}$		
No. spermatophores /female	5♀x5♂	10♀x10♂	20♀x20♂	5♀x5♂	10♀x10♂	20♀x20♂
			AL_{21}			
0	0.0	0.0	1.2	15.0	5.0	8.8
1	30.0	22.5	27.5	60.0	30.0	18.8
2	40.0	35.0	45.0	25.0	45.0	52.5
3	15.0	32.5	18.8	0.0	12.5	17.5
4	15.0	7.5	6.3	0.0	7.5	2.5
5	0.0	2.5	1.3	0.0	0.0	0.0
			BR_{24}			
0	45.0	22.5	43.8	85.0	62.5	58.8
1	55.0	72.5	51.3	15.0	37.5	41.3
2	0.0	5.0	5.0	0.0	0.0	0.0
3	0.0	0.0	0.0	0.0	0.0	0.0
4	0.0	0.0	0.0	0.0	0.0	0.0
5	0.0	0.0	0.0	0.0	0.0	0.0
			Pooled			
0	22.5	11.3	22.5	50.0	33.8	33.8
1	42.5	47.5	39.4	37.5	33.8	30.0
2	20.0	20.0	25.0	12.5	22.5	26.3
3	7.5	16.3	9.4	0.0	6.3	8.8
4	7.5	3.8	3.1	0.0	3.8	1.3
5	0.0	1.3	0.6	0.0	0.0	0.0

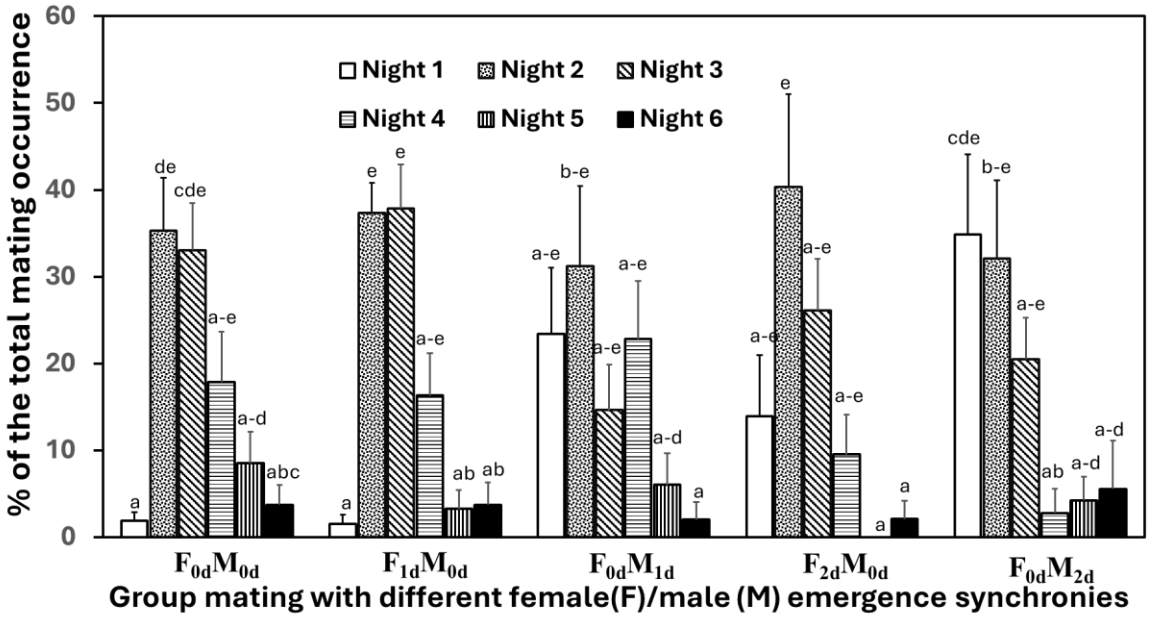

Supporting Information Figure S1. Mating occurrence across six nights under group-mating conditions with different female (F) and male (M) emergence synchronies (Trial II). Percent occurrence (mean ± SEM) for each night was calculated as the number of mating pairs observed during the five nightly observation periods divided by the total number of mating events recorded over all six nights. Replications with two or fewer mating events during the six-night observation period were excluded from the data analysis. Mean values sharing a same letter are not significantly different (ANOVA, Tukey's HSD, $\alpha = 0.05$). Only the first and the last letters are presented over the bar if four or more letters were needed for the HSD tests. For example, the label of a-e over the bar representing the mean of 'Night 5' of $F_{0d}M_{0d}$ means abcde.
